# Dual energy for pulmonary vein isolation using focal ablation technology integrated with a three-dimensional mapping system: SmartfIRE 12-month results

**DOI:** 10.1093/europace/euaf174

**Published:** 2025-08-12

**Authors:** Tom De Potter, Daniel Scherr, Helmut Pürerfellner, Gediminas Račkauskas, Jim Hansen, Johan Vijgen, Thomas Phlips, Sebastien Knecht, Gabor Szeplaki, Hugo Van Herendael, Mads Brix Kronborg, Benjamin Berte, Martin Ruwald, Georgios Kollias, Peter Lukac, Tiffany Tan, Mattias Duytschaever

**Affiliations:** Cardiovascular Center, AZORG, Moorselbaan 164, 9300 Aalst, Belgium; Division of Cardiology, Department of Internal Medicine, Medical University of Graz, Graz, Austria; Department of Cardiology and Electrophysiology, Ordensklinikum Linz Elisabethinen, Linz, Austria; Vilnius University Hospital, Santaros Klinikos, Vilnius University, Vilnius, Lithuania; Department of Cardiology, Gentofte Hospital, University of Copenhagen, Gentofte, Denmark; Cardiology Department, Jessa Hospitals, Hasselt, Belgium; Cardiology Department, Jessa Hospitals, Hasselt, Belgium; Department of Cardiology, AZ Sint-Jan Hospital, Bruges, Belgium; Heart & Vascular Centre, Mater Private Hospital, Dublin, Ireland; Cardiovascular Research Institute, Royal College of Surgeons, Dublin, Ireland; Department of Cardiology, Ziekenhuis Oost-Limburg, Genk, Belgium; Department of Cardiology, Aarhus University Hospital, Aarhus, Denmark; Heart Center, Hirslanden St Anna, Lucerne, Switzerland; Department of Cardiology, Gentofte Hospital, University of Copenhagen, Gentofte, Denmark; Department of Cardiology and Electrophysiology, Ordensklinikum Linz Elisabethinen, Linz, Austria; Department of Cardiology, Aarhus University Hospital, Aarhus, Denmark; Department of Biostatistics and Data Management, Biosense Webster, Inc., Part of Johnson & Johnson MedTech, Irvine, CA, USA; Department of Cardiology, AZ Sint-Jan Hospital, Bruges, Belgium

**Keywords:** Pulsed field ablation, Dual energy, Pulmonary vein isolation, Contact force catheter, Ablation index

## Abstract

**Aims:**

The multicentre, single-arm SmartfIRE study assessed the safety and effectiveness of the novel dual-energy THERMOCOOL SMARTTOUCH SF (DE STSF) contact-force sensing catheter with multimodality generator to deliver radiofrequency (RF) and unipolar biphasic pulsed field (PF) ablation. Three-month follow-up showed a 100% acute success rate with an acceptable safety profile. Results at 12 months postablation are summarized here.

**Methods and results:**

Patients with symptomatic paroxysmal atrial fibrillation underwent pulmonary vein isolation with the recommendation of PF ablation at posterior/inferior and RF ablation at the anterior/ridge/carina segments. The 12-month effectiveness endpoint was freedom from documented symptomatic and asymptomatic atrial arrhythmia on or off antiarrhythmic therapy (assessed by electrocardiogram, remote arrhythmia monitoring, and 24-h Holter), including acute procedural failures. Safety was assessed as the incidence of serious adverse events (SAEs) related to device and/or procedure. Quality of life was evaluated via Atrial Fibrillation Effect on Quality-of-Life (AFEQT) scores, and healthcare utilization was assessed as hospitalization for cardiovascular events and antiarrhythmic drug (AAD) use. Of 149 patients enrolled, 140 had the study catheter inserted (safety population analysis set), and 136 met the eligibility criteria and had ablation energy delivered (per-protocol analysis set). Freedom from symptomatic and asymptomatic atrial arrhythmia at 12 months was 71.5% (84.2% when using standard-of-care monitoring only). The clinical success rate (freedom from symptomatic arrhythmia) was 86.4%, and single procedural success was 81.0% (*n* = 136). The rate of device- and/or procedure-related SAEs was 3.6% (5/140 patients; two cardiac tamponades, two pulmonary vein stenosis, one anaphylactic shock). At 12 months, the overall AFEQT score increased by a median 26.9 points vs. baseline. Cardiovascular hospitalization rate reduced from 20.1 to 11.9% during the 12 months before vs. after ablation, respectively. The use of Class I/III AAD decreased from 60.3% at baseline to 23.9% at 6–12 months postablation. *Post hoc* analysis showed that patients with high adherence to recommended inter-tag distance and PF/RF index during ablation (*n* = 47) had a 12-month freedom from atrial arrhythmia recurrence of 86.9%, while the remaining patients (*n* = 88) had a rate of 64.0%.

**Conclusion:**

The 12-month follow-up of the SmartfIRE study demonstrated the effectiveness, safety, and healthcare benefits of ablation using the DE STSF platform.

**Clinical Trial Registration:**

ClinicalTrials.gov Identifier: NCT05752487

(https://clinicaltrials.gov/study/NCT05752487)

## Graphical Abstract

**Graphical Abstract:**
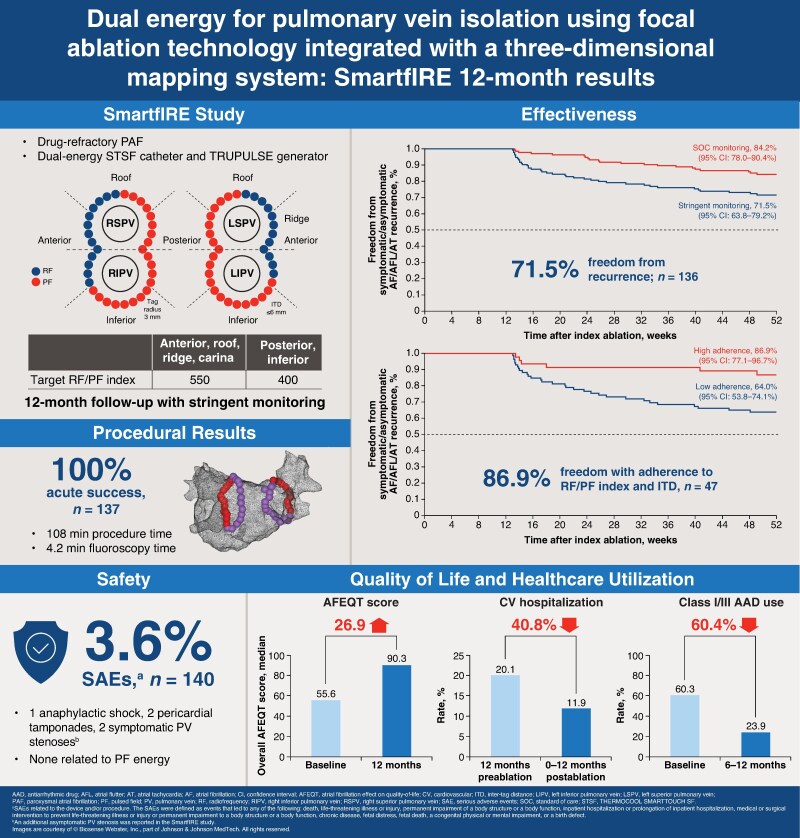

What’s new?

- The SmartfIRE 12-month follow-up showed a favourable effectiveness and acceptable safety profile for this integrated dual-energy ablation system.
- Patients with higher adherence to the recommended ablation workflow had a higher rate of 12-month freedom from atrial arrhythmia recurrence compared with patients with lower workflow adherence.
- A clinically meaningful improvement in quality of life and reduced cardiovascular hospitalization and antiarrhythmic drug use were seen at 12 months after ablation compared with the baseline.

## Introduction

Pulsed field (PF) ablation (PFA) is a newer ablation technology for the treatment of atrial fibrillation (AF) with demonstrated safety and effectiveness in multiple studies.^1–4^ Compared with thermal ablation, such as radiofrequency (RF) and cryoablation, PFA has a more tissue-selective mechanism, resulting in a reduced risk of collateral tissue damage and associated complications, such as pulmonary vein (PV) stenosis or atrioesophageal fistula, while maintaining treatment effectiveness.^5–7^ Despite these potential advantages, in clinical practice, thermal ablation remains the predominant mode of energy delivery for AF ablation,^8^ highlighting the need for more research investigating the clinical benefits associated with PFA.

The contact force (CF)–sensing dual-energy THERMOCOOL SMARTTOUCH SF (DE STSF) catheter with multimodality generator TRUPULSE (Biosense Webster, Inc., part of Johnson & Johnson MedTech, Irvine, CA, USA) incorporates both the tissue selectivity provided by PFA and the properties of RF while maintaining a familiar focal ablation technology. Equipped with the magnetic sensor, the DE STSF catheter is fully integrated with the 3D navigation system (CARTO 3; Biosense Webster, Inc.), including both the PF index and RF SURPOINT index, and thus optimizes effectiveness while minimizing fluoroscopy time. The catheter was developed with the aim of overcoming certain limitations of using PFA alone, such as difficulties or risks in ablation near conduction tissue or coronary arteries. Preclinical findings with this catheter and generator showed similar lesion sizes with PF vs. RF delivery, with PFA producing more mature scar formation compared with RF ablation, as well as reduced chronic inflammation and myocardial necrosis.^9^ The RF index was developed by incorporating CF, power, and time, based on the experimental work by Nakagawa *et al.*,^10,11^ and this index predicted lesion depth in the canine ventricle and atrium with high accuracy. In a clinical setting, minimum index values of 400 posteriorly and 550 anteriorly were required to prevent acute PV reconnections^12,13^ ; these index values were prospectively evaluated in multiple large studies, demonstrating consistent safety and effectiveness outcomes.^13–15^ In preclinical experiments of PF applications using a focal STSF catheter, CF and number of PF applications were strongly associated with lesion depth.^16^ A logarithmic formula of the PF index incorporating these variables was developed, and it was validated in a prospective swine model, with 100% accuracy in lesion depth prediction (±1.5 mm).^17^ Additionally, the combination of PF and RF further enhances lesion penetration,^18^ highlighting the benefits of the DE STSF ablation platform in achieving transmural lesions.

The SmartfIRE study was a prospective, multicentre, single-arm trial conducted in Europe to assess the clinical safety and effectiveness of this novel dual-energy integrated technology for the treatment of patients with drug-refractory, symptomatic paroxysmal AF. The 3-month safety and effectiveness findings were reported previously and showed a 100% acute procedural success rate and an acceptable safety profile.^19^ Here, we present outcomes at 12 months of follow-up.

## Methods

### Study design and population

The design of the SmartfIRE study (NCT05752487) has been described previously.^19^ In brief, this prospective, multicentre study evaluated the safety and effectiveness of the DE STSF catheter in combination with the TRUPULSE generator and the CARTO 3 mapping system. Details of study sites and investigators are provided in Supplementary material online, *Table S1*.

Eligible patients were adults who were 18–75 years of age, had diagnosed symptomatic paroxysmal AF, had previously failed or did not tolerate ≥1 antiarrhythmic drug (AAD; Classes I–IV), and had a clinical indication for catheter ablation by PV isolation (PVI). Full inclusion and exclusion criteria are shown in Supplementary material online, *Table S2*.

Ethics committees at all participating sites and national authorities in the participating countries reviewed and approved the SmartfIRE study, which was conducted in accordance with the International Conference on Harmonization Good Clinical Practices and the Declaration of Helsinki. Written informed consent was provided by all patients prior to treatment in the study.

### Ablation procedure and follow-up

The ablation platform (DE STSF catheter with TRUPULSE generator and CARTO 3 mapping system) and study ablation procedure have been described previously.^9,16,19^ Pulmonary vein isolation was performed with focal ablation to obtain a contiguous lesion set for ipsilateral PVs, with PFA recommended at posterior/inferior segments and RF ablation recommended at anterior/ridge/carina segments. Based on the existing RF index clinical evidence^14,20,21^ and PF index validation work by Nakagawa *et al.*,^16,17^ in this study, a target index of 550 for anterior, roof, ridge, and carina and a target index of 400 for posterior and inferior were recommended, regardless of energy type. The recommended ablation workflow also included a tag size of 3 mm and an inter-tag distance (ITD) ≤ 6 mm. The deflectable VIZIGO sheath (Biosense Webster, Inc.) was used at physicians’ discretion. Pulmonary vein isolation (entrance block) confirmation was performed following adenosine/isoproterenol challenge with no waiting period. As needed, acute reconnections were treated with additional applications of PF/RF energy. Cavotricuspid isthmus (CTI) ablation was permitted with documented typical atrial flutter (AFL) using RF or PF energy. One to 2 mg of intravenous or intracoronary nitroglycerin was recommended for PFA near the coronary artery, such as for CTI ablation. Ablation was followed by a 3-month blanking period. As previously reported,^19^ a prespecified subset of 30 patients underwent electroanatomic remapping at a mean of 79.3 ± 6.9 days postindex procedure; reisolation was performed for any reconnections, with the same recommended ablation workflow. A repeat ablation procedure during the follow-up period did not reset the blanking period.

Monitoring of atrial arrhythmia recurrence during the follow-up evaluation period included 12-lead electrocardiogram monitoring [preprocedure, predischarge, and at Months 1, 3, 6, and 12 visits, as well as unscheduled visits (if any)], 24-h Holter monitoring (at Months 3, 6, and 12), and remote arrhythmia monitoring [transtelephonic monitoring (TTM); weekly between Months 1 and 5, monthly between Months 6 and 12, and following any symptomatic episodes, recorded for a duration of 1 min].

### Effectiveness and safety endpoints

The primary effectiveness endpoint (acute procedural success) and primary safety endpoint [incidence of primary adverse events (PAEs)] were reported previously.^19^ All 12-month endpoints reported here were considered secondary or additional endpoints of the SmartfIRE study.

The 12-month effectiveness endpoint was defined as freedom from documented symptomatic and asymptomatic atrial arrhythmia [AF, AFL, or atrial tachycardia (AT) of unknown origin] episodes of ≥30 s on an arrhythmia monitoring device during the effectiveness evaluation period (Days 91–365) on or off AAD therapy. Acute procedural failures were also considered failures for this endpoint. The performance goal of this endpoint was set at 50%. The 12-month freedom from documented atrial arrhythmia (AF/AFL/AT) was also calculated after excluding the remote arrhythmia monitoring (i.e. using the standard-of-care monitoring only).

Safety was assessed over the 12-month period based on the incidence of serious adverse events (SAEs) related to the device and/or the procedure. The SAEs were defined if they led to any of the following: death, life-threatening illness or injury, permanent impairment of a body structure or a body function, in-patient hospitalization or prolongation of patient hospitalization, medical or surgical intervention to prevent life-threatening illness or injury or permanent impairment to body structure or a body function, chronic disease, fetal distress, fetal death, or a congenital physical or mental impairment or birth defect.

Additional effectiveness endpoints were evaluated. Clinical success was defined as freedom from documented symptomatic AF/AFL/AT recurrence during the effectiveness evaluation period (acute procedure failure was also considered a failure mode of this 12-month symptomatic recurrence free endpoint). Single procedural success was defined as freedom from documented symptomatic AF/AFL/AT episodes during the effectiveness evaluation period following a single index ablation procedure. A composite endpoint evaluated was the freedom from documented AF/AFL/AT recurrence during the effectiveness evaluation period plus any of the additional failure modes, including failure to achieve acute procedural success, taking a new Class I/III AAD or a previously failed Class I/III AAD at a greater than the highest ineffective historical dose during the effectiveness evaluation period, having >1 repeat ablation in the blanking period or any repeat ablation during the effectiveness evaluation period. Freedom from repeat ablation procedures for left atrial arrhythmia within the 12-month follow-up period was also evaluated.

Patient quality of life was assessed via Atrial Fibrillation Effect on Quality-of-Life (AFEQT) scores at baseline and at 3, 6, and 12 months after the ablation procedure. Healthcare utilization was assessed by comparing the hospitalization rates for cardiovascular events during the 12-month follow-up period with the 12 months prior to baseline and the use of AADs at baseline and at 6–12 months postablation.

### Statistical methods

The safety population analysis set included all enrolled patients with insertion of the study catheter, irrespective of energy delivery, and was used in the current analysis to assess the occurrence of SAEs. The modified intent-to-treat (mITT) analysis set, which was used to assess the occurrence of PAEs for the 3-month analyses, included enrolled patients who met eligibility criteria and had insertion of the study catheter. The per-protocol (PP) analysis set, which was used to evaluate the 12-month effectiveness endpoints, included patients who underwent PF/RF ablation via the study ablation platform, were treated for the study-related arrhythmia, and had no major protocol deviations that would affect the integrity of the safety and effectiveness data.

The Kaplan–Meier estimates of the 12-month success rates were generated separately for the effectiveness endpoints. The two-sided 95% confidence interval (CI) for the success rate was estimated using Greenwood’s formula. A *post hoc* subgroup analysis was performed using the Kaplan–Meier estimates for the 12-month success rate based on levels of adherence to the recommended ITD and PF/RF index in index ablation procedures. The high adherence group consisted of patients with >95% of applications maintaining an ITD of ≤6 mm and over 70% of applications achieving a minimum PF/RF index of 400 in the posterior wall and 500 in the anterior wall of the PV regions. Patients not meeting these criteria were categorized into the low adherence group. Cox proportional hazards regression models were used to estimate the adjusted hazard ratios of 12-month AF recurrence in the high and low adherence groups, adjusting for potential confounders. Important factors, such as age, sex, AF history, LA diameter, LA volume, total number of valid PF/RF applications, and total PV ablation time were considered as potential confounders in the analysis. All the continuous variables were categorized by quartiles in the model. As these analyses involved cut-offs that were not prespecified in the protocol for the grouping of adherence, the reported *P* values should be interpreted with caution and considered hypothesis-generating rather than confirmatory.

The baseline characteristics, safety endpoints, AFEQT score, and cardiovascular hospitalization were summarized descriptively. The McNemar test was employed to determine if there is a significant difference in the proportion of patients using all classes of AAD or Class I/III AADs at baseline and 6–12 months after the ablation procedure. The Wilcoxon rank-sum test was used to compare procedural parameters in patients undergoing ablation procedures with or without the VIZIGO sheath, including total procedure duration and fluoroscopy time.

All statistical analyses were performed using SAS 9.4 or SAS Studio 3.8 (SAS Institute Inc., Cary, NC, USA).

## Results

A total of 149 patients were enrolled in the SmartfIRE study. Enrolment and participant characteristics for the primary safety and effectiveness analyses were reported previously.^19^ During the effectiveness evaluation period, an additional participant was found not to meet study eligibility criteria and was therefore excluded from the mITT and PP analysis sets. Consequently, for this 12-month analysis, the safety population remained at 140 patients, while the mITT analysis comprised 137 patients, and the PP analysis comprised 136 patients. Updated patient disposition and procedural characteristics are displayed in Supplementary material online, *Figure S1* and Supplementary material online, *Table S3*. An overall compliance rate of 84.6% for TTM was observed in the PP analysis set. There were 52 patients in which the VIZIGO sheath was used. Both the procedure time and fluoroscopy time were significantly shorter in patients with VIZIGO compared with those without it (see Supplementary material online, *Table S4*).

### Effectiveness

**Figure 1:**
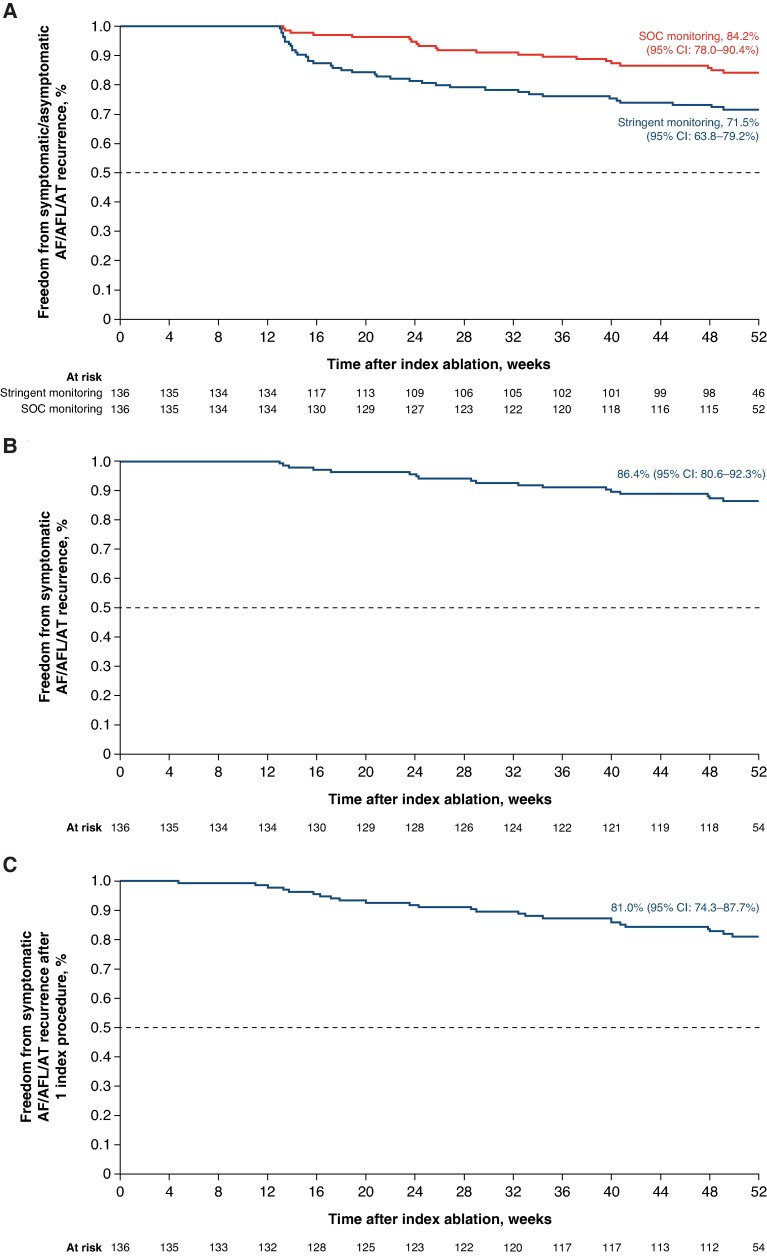
Kaplan–Meier analysis of (*A*) freedom from documented symptomatic or asymptomatic AF/AT/AFL recurrence, (*B*) freedom from acute procedural failure or documented symptomatic AF/AT/AFL recurrence (clinical success), (*C*) freedom from acute procedural failure or documented symptomatic AF/AT/AFL recurrence following a single index ablation procedure. Per-protocol analysis set, *n* = 136. AF, atrial fibrillation; AFL, atrial flutter; AT, atrial tachycardia; CI, confidence interval; SOC, standard of care. Images are courtesy of © Biosense Webster, Inc., part of Johnson & Johnson MedTech. All rights reserved.

At 12-month follow-up, 38 patients had documented recurrences, while two patients did not have the 12-month follow-up data in the PP analysis set. The recurrences consisted of 34 cases of AF, four cases of AFL, and zero case of AT. There were no acute procedure failures.^19^ Therefore, 96 patients (71.6%, 96/134) were free from documented symptomatic and asymptomatic atrial arrhythmia recurrence, with the lower bound of the two-sided exact 95% CI at 63.2%, indicating that the performance goal was met. The Kaplan–Meier analysis indicated that freedom from documented symptomatic and asymptomatic AF/AFL/AT recurrence was 71.5% (95% CI: 63.8–79.2%; *Figure 1A*). When accounting for arrhythmia detected without use of TTM (i.e. standard-of-care monitoring), freedom from documented symptomatic and asymptomatic AF/AFL/AT recurrence was 84.2% (95% CI: 78.0–90.4%; *Figure 1A*).

A total of 118 patients in the PP analysis set were free of symptomatic atrial arrhythmia recurrence at Month 12 (i.e. clinical success). The clinical success rate by the Kaplan–Meier analysis was 86.4% (95% CI: 80.6–92.3%; *Figure 1B*). A total of 111 patients were free from symptomatic recurrences following one single ablation procedure. The Kaplan–Meier estimate of freedom from symptomatic AF/AFL/AT recurrence after a single procedure was 81.0% (95% CI: 74.3–87.7%; *Figure 1C*).

Forty-one patients experienced at least one failure mode of the composite endpoint (atrial arrhythmia recurrence, acute failure, new or higher Class I/III AAD, or repeat ablation). The Kaplan–Meier rate of freedom from the composite failure modes was 69.3% (95% CI: 61.4–77.1%).

### Safety

Over the 12-month follow-up, five SAEs related to the device and/or procedure were observed in five patients in the safety analysis set (5/140, 3.6%), comprising two cardiac tamponade events, two cases of PV stenosis, and one case of anaphylactic shock. The two pericardial tamponades both occurred on the day of the ablation procedure and were attributed to difficult transeptal punctures, requiring pericardiocentesis or surgery.^19^ One patient developed anaphylactic shock to adenosine on the day of the procedure; the patient recovered after medication, and the event was assessed by the investigator as related to the study procedure but not related to the study catheter or generator. One patient was diagnosed with PV stenosis in the left superior PV (LSPV) and left inferior PV (LIPV) 370 days postprocedure after persistent dyspnoea, requiring inpatient hospitalization and PV dilatation; the event resolved and was assessed by the investigator as related to RF ablations performed into the ostia of vein during the index ablation procedure. The other serious PV stenosis was first observed at 2 months (in LIPV) after the index ablation and was then treated with dilation and stenting. In this case, multiple overlapping RF lesions with high ablation index values and application of RF lesions inside the PV ostium were found to be contributing factors.^19^ All of these SAEs resolved without clinical sequelae, and none were related to PF energy. The summary of SAEs related to the procedure and/or device and PAEs is shown in *Table 1*. Another instance of asymptomatic LSPV stenosis identified at the prespecified 3-month cardiac computed tomography (CT) angiography was previously described.^19^ This was found to be related to high RF index and overlapping RF applications. This patient was asymptomatic and did not require any intervention; therefore, the SAE criteria were not met (see Supplementary material online, *Figure S2*). No unanticipated adverse events related to procedure and/or device were reported. No adverse events caused by muscle contraction were observed.

**Table 1:** Summary of SAEs and PAEs

Cases	SAEs^a^	PAEs^b^	Note
Anaphylactic shock	Yes	No	Received medication and recovered
Pericardial tamponade	Yes	Yes	Pericardial puncture
Pericardial tamponade	Yes	Yes	Surgery
PV stenosis	Yes	Yes	Symptomatic, required stenting
PV stenosis	Yes	No	Symptomatic, required PV dilation
PV stenosis	No	Yes	Asymptomatic, no intervention
Stroke/CVA	No	Yes	No additional treatment, no neurological sequelae
Pericarditis	No	Yes	Resolved with medication
**Total**	5	6	

CVA, cerebrovascular accident; mITT, modified intent-to-treat; PAE, primary adverse event; PV, pulmonary vein; SAE, serious adverse event.

^a^SAEs related to the device and/or procedure. The SAEs were reported at 12 months in the safety population analysis set, *n* = 140.

^b^PAEs were adjudicated by the Clinical Events Committee and were reported at 3 months in the mITT analysis set, *n* = 137.^19^ The PAEs included major vascular access complication or bleeding, myocardial infarction, pericarditis, pulmonary oedema, stroke or CVA, transient ischaemic attack, thromboembolism, heart block, vagal nerve injury or gastroparesis, cardiac tamponade or perforation (up to 30 days post-procedure), and permanent phrenic nerve paralysis as well as PV stenosis, atrioesophageal fistula, and death (up to 90 days post-procedure).

### Repeat procedures

**Figure 2:**
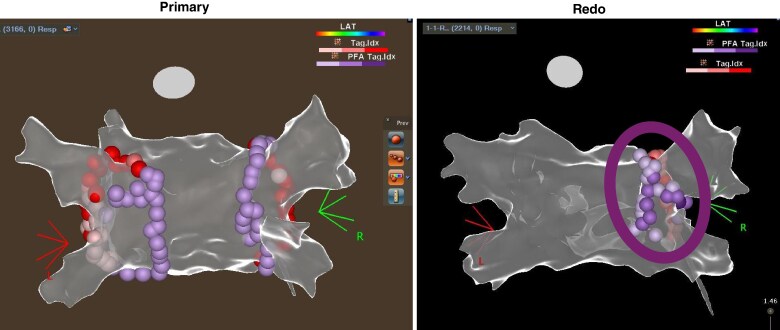
Example of repeat procedure. Left: index procedure. Right: repeat procedure with additional carinal ablation performed. LAT, local activation time; PFA, pulsed field ablation. Images are courtesy of © Biosense Webster, Inc., part of Johnson & Johnson MedTech. All rights reserved.

Overall, 18 patients underwent repeat ablation procedures. The Kaplan–Meier rate of freedom from repeat ablation for AF/AFL/AT recurrence was 86.4% (95% CI: 80.5–92.2%) in the PP analysis set. Of the 18 patients who underwent repeat ablations, PV reconnection was seen in 14 patients and in 31 veins (31/67, 46.3%). The distributions of PV reconnections were 1/1 (100%) at the right common PV, 10/17 (58.8%) at the right superior PV, 8/17 (47.1%) at the right inferior PV, 6/14 (42.9%) at LSPV, 5/14 (35.7%) at LIPV, and 1/4 (25%) at the left common PV. Most of the PV reconnections were located at the carina segments (23/38, 60.5%); in 13 patients (13/18, 72.2%), the posterior carina of the right circle was involved. These reconnections were seen at sites of both PF and RF lesions, with no significant differences. Case review indicated that sites of reconnections can be independently associated with lack of stability in that area, not reaching target index, and/or having lesion gaps of >6 mm. Some cases required a carina line, which was not performed in the index procedure (*Figure 2*). In seven (38.9%) patients, non-PV lesions were required.

Among the 18 patients with durable PVI at the protocol-mandated remapping procedure,^19^ five recurrences were documented, and one recurrence was observed among the group of 12 patients with PV reconnections of this subset.

### Quality of life and healthcare utilization

**Figure 3:**
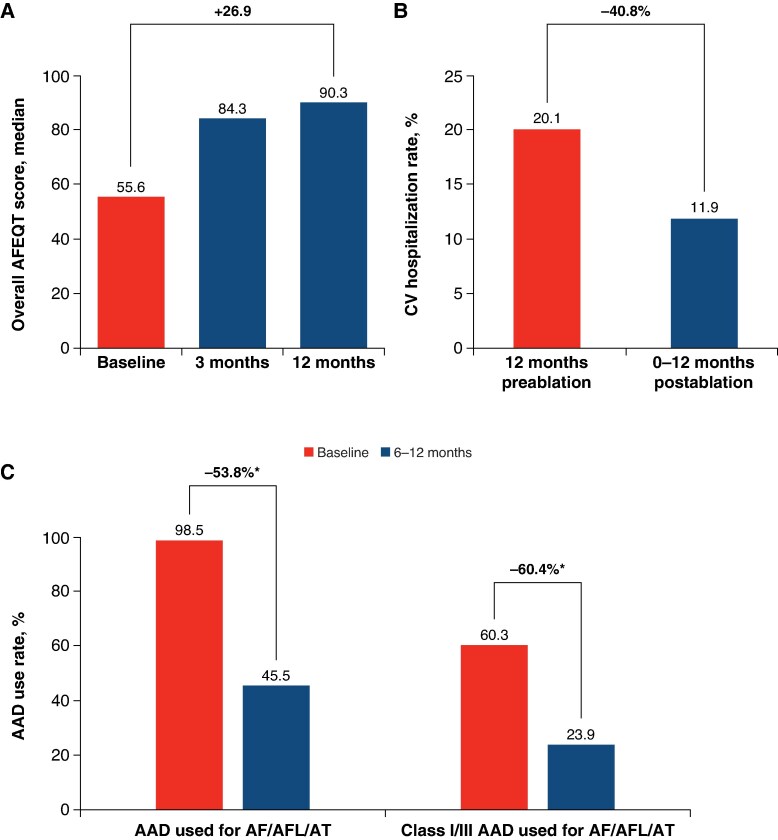
Quality of life and healthcare utilizations. (*A*) AFEQT score, (*B*) cardiovascular hospitalizations, and (*C*) AAD use. Per-protocol analysis set. AAD, anti-arrhythmic drug; AF, atrial fibrillation; AFEQT, Atrial Fibrillation Effect on Quality-of-Life; AFL, atrial flutter; AT, atrial tachycardia; CV, cardiovascular. *n* = 136 for the AFEQT score; *n* = 134 for CV hospitalization; for AAD use, *n* = 136 at baseline and 134 at 6–12 months. *P < 0.001 with McNemar test. Images are courtesy of © Biosense Webster, Inc., part of Johnson & Johnson MedTech. All rights reserved.

At 12 months, the overall AFEQT score increased by a median (Q1, Q3) of 26.9 (11.1, 42.6) points compared with baseline (*Figure 3A*). Similar improvements were observed across all AFEQT domain scores of AF-related symptoms, daily activities, treatment concerns, satisfaction with current treatment control, and satisfaction with relief of symptoms.

During the 12 months of follow-up, 16 patients (11.9%) had hospitalizations related to cardiovascular events compared with 27 out of 134 patients (20.1%) 12 months prior to study enrolment, representing an absolute reduction of 8.2% and a 40.8% relative reduction in cardiovascular hospitalization rates in the PP analysis set (*Figure 3B*). At 6–12 months after ablation, overall and Class I/III AADs were used in 45.5% (61/134) and 23.9% (32/134) patients, respectively, resulting in a relative reduction of 53.8% in all and 60.4% in the use of Class I/III AADs compared with baseline (*P* < 0.001; *Figure 3C*).

### Workflow analysis

**Figure 4:**
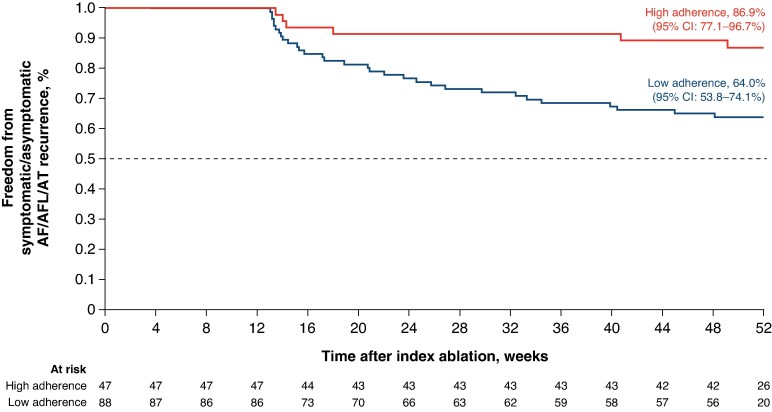
Twelve-month outcome stratified by workflow adherence. Per-protocol analysis set, *n* = 135. AF, atrial fibrillation; AT, atrial tachycardia; AFL, atrial flutter; CI, confidence interval. Images are courtesy of © Biosense Webster, Inc., part of Johnson & Johnson MedTech. All rights reserved.

*Post hoc* subgroup analyses were conducted to assess the potential impact of high adherence to the recommended ITD and PF/RF index values in the ablation procedure on the 12-month effectiveness outcomes. The Kaplan–Meier estimate for the 12-month success rate of freedom from documented AF/AFL/AT recurrence was 86.9% (95% CI: 77.1–96.7%) in the high adherence group, where >95% of applications maintained an ITD of ≤6 mm, and >70% of applications reached PF/RF index values of 400 posteriorly and 500 anteriorly per patient. In contrast, the 12-month success rate in the low adherence group was 64.0% (95% CI: 53.8–74.1%; *n* = 88; *Figure 4*). The mean percentage of applications exceeding these cut-off values was lower in the anterior region, at 66.8 ± 16.7%, compared with 85.4 ± 12.1% in the posterior region. In all the study data, a high proportion (93.6 ± 9.6%) of applications in the posterior region complied with the recommendation to use PF energy, and the adherence percentage to RF energy recommendation was lower in the anterior region (78.8 ± 16.1%). The hazard of recurrence at 12 months was 0.31 (95% CI: 0.13–0.74), which was significantly lower in patients in the high adherence group compared with those in the low adherence group (*P* = 0.008). The adjusted hazard ratio for 12-month recurrence in the high adherence group vs. the low adherence group was 0.21 (95% CI: 0.09–0.51), as determined from analyses adjusted for the number of valid PF/RF applications and sex (*Table 2*).

**Table 2:** Cox model outcome

Parameter	Hazard ratio (95% CI)	*P* value^a^
High adherence group vs. low adherence group	0.21 (0.09–0.51)	0.001
Sex: female vs. male	2.49 (1.28–4.84)	0.007
Number of valid PF/RF applications	**−**	0.004
Number of PF/RF applications: 59–69 vs. ≤59	1.38 (0.55–3.43)	**−**
Number of PF/RF applications: 69–82 vs. ≤59	2.57 (1.07–6.15)	**−**
Number of PF/RF applications: > 82 vs. ≤59	0.34 (0.10–1.15)	**−**

CI, confidence interval; PF, pulsed field; RF, radiofrequency; vs., versus.

^a^ *P* value for Type 3 test.

## Discussion

The 12-month results of the SmartfIRE study demonstrated long-term effectiveness and safety of the DE STSF ablation platform for the treatment of paroxysmal AF, with a 71.5% rate of freedom from AF/AFL/AT recurrence with stringent monitoring and a 3.6% rate of device and/or procedure-related SAEs. These results add to those observed during the 3-month follow-up in the SmartfIRE study.^19^

Our findings should be viewed in light of recent technological advances in AF ablation,^22^ including the 3D electro-anatomical integration, the introduction of CF-sensing catheters, and the emergence of PFA. Compared with earlier technologies, 3D mapping–integrated, index-guided focal DE ablation offers improvement in efficacy and procedural efficiency and is an example of continued advancement in ablation technology and technique, which may partly explain the favourable outcomes observed in the study.

The 12-month effectiveness seen in this study was similar to that previously reported in the inspIRE (75.6%), admIRE (75.4%), and PULSED AF (69.5%) studies of PFA in the treatment of paroxysmal AF with similar stringent monitoring.^23–25^ Additionally, a study using a dual-energy lattice tip catheter reported 78.3% freedom from 1-year recurrence in patients with paroxysmal AF.^26^ However, the study allowed linear ablations in addition to PVI, included various PF waveforms, and analysed only 70 paroxysmal AF patients. These factors make it difficult to directly compare the results. When using standard-of-care monitoring only during the 12-month follow-up, the rate of freedom from AF/AFL/AT recurrence observed in the current study was 84.2%, comparable to the rates reported in inspIRE (85.8%), EU-PORIA (80%), PLEASE-AF (86.7%), and REAL-AF (81.6%), which used PF or RF technologies with similar monitoring methods.^4,23,27,28^ Consistent with these previous findings, the SmartfIRE study demonstrated a favourable 12-month effectiveness outcome.

The study reported five SAEs related to device and/or procedure during the 12-month follow-up. This SAE rate is within the range (0.5–4.9%) observed in other recent studies evaluating PF or dual-energy technologies^1,26,29–31^; however, variations in the definitions of adverse events and differences in evaluation periods across studies may limit the ability to draw direct comparisons. Pulmonary vein stenosis was seen in three patients in this study, one of whom was asymptomatic and detected during protocol-defined cardiac CT/magnetic resonance angiogram imaging for PV narrowing assessment.^19^ Although acute treatments for the symptomatic stenoses were successful, these two patients may need ongoing follow-up due to the high risk of restenosis after stenting.^32^ Pulmonary vein stenosis is a known complication of AF ablation resulting from thermal injury to the PVs with a high risk associated with ablation in the PV ostia.^8,33,34^ This is in agreement with the case review in this study where multiple overlapping RF lesions, high RF ablation index values, and RF ablation inside the PVs were seen. Preclinical data showed that PF energy delivery with the DE STSF catheter and TRUPULSE generator did not reduce PV diameter,^9^ consistent with the tissue-selective effect of PFA.^29,35^ Based on these observations, the PV stenoses were not attributed to PF energy. Additionally, consistent with the current study, previous data have indicated that PV stenosis more frequently occurs in the left PVs than in the right PVs after RFA.^33,36^ It may be associated with anatomic characteristics such as the relatively small diameter of the LIPV and the relatively cranial orientation of the LSPV ostium^36,37^; therefore, RF in the left PV should be performed with an understanding of these anatomical characteristics and adjusted accordingly. Prior studies have reported a higher rate of periprocedural haemolysis with PFA compared with RF ablation^38,39^; however, no haemolysis or acute kidney injury was observed in the SmartfIRE study, although biomarkers of haemolysis or renal tubular damage were not tested.

The 12-month clinical success rate, defined as freedom from documented symptomatic atrial arrhythmia during the effectiveness evaluation period, was 86.4%. In studies that have used the same definition based on stringent monitoring strategy, similar rates were observed in the Q-FFICIENCY study of very high-power short-duration RF ablation (86.0%) and inspIRE (81.7%) and PULSED AF (79.7%) studies using PFA.^23,24,40^ The single-procedure success rate at 12 months in the current study was 81.0%, which compared favourably with that seen in the Q-FFICIENCY study (76.3%).^40^ The freedom from AF/AFL/AT recurrence with additional failure modes at 12 months was 69.3%. Other PFA technologies assessing a composite effectiveness endpoint have reported similar results between 66 and 75%, although the components of the composites differed.^24,25,29,30^

Among the patients who underwent repeat ablation, non-PV targets were ablated in 38.9%. Sites of PV reconnections were primarily at posterior and carina and were associated with lack of catheter stability, large ITD, and low target ablation index in the index procedure. These findings are consistent with the observations at 3-month remapping.^19^ Some cases required a carina line that was not performed during the index procedure. These highlight the need to address this anatomical region in the index procedure with careful attention to stability and access, particularly on the right-sided PVs.^41–43^ Considering the higher variability of tissue thickness in the posterior carina and the proportion of reconnections observed in this region, the use of RF energy with a higher target index and the stability indication may improve transmurality and should be studied in the future.

The current study demonstrated a clinically meaningful improvement in quality of life, as shown by a median increase in the AFEQT score of 26.9 points. A ‘clinically meaningful increase’ in AFEQT score is estimated to be between 5 and 19 points, with the latter representing a conservative estimate, widely accepted as corresponding to a substantial and meaningful improvement in quality of life.^44,45^ Meaningful improvements were also seen across the AFEQT domain scores of symptoms, daily activities, treatment concerns, and treatment satisfaction, which were observed starting at the 3-month assessment and maintained through 12 months of follow-up.

At 12 months after catheter ablation using the study device, the rate of hospitalizations for cardiovascular causes in our study population was reduced by 40.8% compared with preablation. Similarly, the VISTAX study showed a reduction of 37.3% in the number of patients who had any cardiovascular hospitalization.^46^ In the admIRE study, in which the study cohort had a lower rate of cardiovascular hospitalization before enrolment (3.3% over 6 months), the rate decreased to 0 during the 6- to 12-month period postablation.^25^ In the current study, significant reduction in the use of all and Class I/III AADs for management of atrial arrhythmia was seen at 12 months post-ablation. These demonstrate the beneficial effect of ablation using the study devices on healthcare utilization with potential impact of health economic outcomes.

It has been repeatedly shown that an RF index-guided approach improves the 1-year outcome of AF ablation,^15,47^ but the impact of using PF index or with dual-energy ablation is yet to be explored. Pulsed field index (incorporating CF and number of PF applications) and RF index (VISTAG SURPOINT; incorporating CF, power, and time) are important parameters in the DE STSF ablation platform for energy delivery and lesion formation.^16,20^ In this study, a target RF/PF index and ITD was recommended with a goal of delivering a transmural and contiguous lesion set. The *post hoc* exploratory analysis showed that, in patients with greater adherence to the recommended workflow, a higher rate of freedom from recurrence was achieved. It should be noted that the majority of the index procedures did not fully align with the recommended ablation workflow. It is because a lower than cut-off index was achieved in instances when PF was used anteriorly, such as after phrenic capture and at the roof. Although the study showed a low acute reconnection rate after adenosine/isoproterenol challenge,^19^ the long-term data suggest a trend towards better outcomes when the target index is achieved. Further investigation is needed to validate and consolidate the findings. Additionally, reconnections seen during the repeat procedures also supported the importance of catheter stability and carina ablations during the index procedure. The lack of respiratory gating and stability indication for PF ablation in the software used in this trial was a limitation. This is expected to improve in clinical practice once updated software with stability indication and respiratory gating is available.^48,49^

### Study limitations and future direction

The limitations of this study include its non-controlled single-arm study design, particularly the lack of prespecified analysis to validate outcomes based on RF/PF index. Further studies designed to assess clinical outcomes based on target ablation index will be needed. Although a rigorous monitoring schedule with high compliance was followed, the failure rate in this study was defined according to the current guideline of a 30-s duration of recurrence. Additional data are needed to determine clinically relevant endpoints with the use of dual-energy technology. Similarly, at the time this study was conducted, a 90-day blanking period was the standard; future studies should consider adopting the updated 60-day blanking period.^8^ Moreover, the study design did not incorporate continuous intra-cardiac monitoring; therefore, AF burden cannot be assessed. Additionally, this study investigated the performance of the technology in PVI; future studies evaluating the feasibility and outcomes in other lesion sets may help determine energy selection for specific targets using such a focal dual-energy catheter. Finally, as the first clinical use of a new ablation technology, the DE STSF platform has room for improvement in terms of algorithm and efficiency, which will be further explored as experience with the platform grows. Future studies utilizing newer CARTO software will provide more accurate data on stability.

## Conclusion

This 12-month follow-up of the SmartfIRE study confirmed the effectiveness, safety, quality of life, and healthcare benefits of PV ablation using the DE STSF platform, in patients with paroxysmal AF, providing a 71.5% freedom from atrial arrhythmias at 12 months, along with a low rate of device- and/or procedure-related SAEs, clinically meaningful improvements in quality of life, and a reduction in cardiovascular hospitalizations and AAD use. In this study, higher adherence to the recommended ablation workflow was associated with higher effectiveness, but further studies are still required.

## Supplementary Material

euaf174_Supplementary_Data

## Data Availability

Johnson & Johnson MedTech has an agreement with the Yale Open Data Access (YODA) Project to serve as the independent review panel for the evaluation of requests for clinical study reports and patient-level data from investigators and physicians for scientific research that will advance medical knowledge and public health. Requests for access to the study data can be submitted through the YODA Project site at http://yoda.yale.edu.
