# Supplementary material for: Dual energy for pulmonary vein isolation using focal ablation technology integrated with a three-dimensional mapping system: SmartfIRE 12-month results
Source: Europace. 2025 Aug 12;27(9):euaf174. doi: 10.1093/europace/euaf174 (PMC12448222; doi:10.1093/europace/euaf174)
Supplement: euaf174_Supplementary_Data [file euaf174_supplementary_data.docx]

**SUPPLEMENTARY MATERIALS**

**Supplemental Table S1. Study sites and principal investigators.**

| **Study site** | **Principal investigator** |
| --- | --- |
| Aarhus University Hospital, Aarhus, Denmark | Peter Lukac |
| AZ Sint-Jan Hospital, Bruges, Belgium | Mattias Duytschaever |
| Cardiovascular Center, AZORG, Aalst, Belgium | Tom De Potter |
| Gentofte Hospital, University of Copenhagen, Gentofte, Denmark | Jim Hansen |
| Jessa Hospitals, Hasselt, Belgium | Johan Vijgen |
| Medical University of Graz, Graz, Austria | Daniel Scherr |
| Ordensklinikum Linz Elisabethinen, Linz, Austria | Helmut Pürerfellner |
| Vilnius University Hospital, Santaros Klinikos, Vilnius University, Vilnius, Lithuania | Gediminas Račkauskas |
| Ziekenhuis Oost-Limburg, Genk, Belgium | Hugo Van Herendael |

The study sites are listed in alphabetical order.

**Supplemental Table S2. Patient inclusion and exclusion criteria.**

| **Inclusion criteria**  (Candidates for this study must meet ALL of the following criteria) | - Diagnosed with symptomatic paroxysmal AF defined as AF that terminates spontaneously or with intervention within 7 days of onset. This paroxysmal AF was considered to be symptomatic if symptoms related to AF were experienced by the patient. - Selected for AF ablation procedure by PVI. - Failed ≥1 AAD (Class I to IV) as evidenced by recurrent symptomatic AF, or intolerable or contraindicated to the AAD. - Age 18-75 years. - Willing and capable of providing consent. - Able and willing to comply with all pre-, post- and follow-up testing and requirements. |
| --- | --- |
| **Exclusion criteria**  (Candidates were excluded if ANY of the following criteria applied) | - Previously known AF secondary to electrolyte imbalance, thyroid disease, or reversible or noncardiac cause (eg, documented obstructive sleep apnea, acute alcohol toxicity, morbid obesity [BMI >40 kg/m²], renal insufficiency [with an estimated creatinine clearance <30 mL/min/1.73 m^2^]). - Previous LA ablation or surgery. - Patients known to require ablation outside the PV region (eg, atrioventricular reentrant tachycardia, atrioventricular nodal reentry tachycardia, atrial tachycardia, ventricular tachycardia, and Wolff-Parkinson-White). - Previously diagnosed with persistent AF (>7 days in duration). - Severe dilatation of the LA (LAD >50 mm antero-posterior diameter in case of transthoracic echocardiography). - Presence of LA thrombus. - Severely compromised LVEF (<40%). - Uncontrolled heart failure or NYHA Class III or IV. - History of blood clotting, bleeding abnormalities or contraindication to anticoagulation (heparin, warfarin, or dabigatran). - History of a documented thromboembolic event (including TIA) within the past 6 months. - Previous percutaneous coronary intervention / myocardial infarction within the past 2 months. - Previous coronary artery bypass grafting in conjunction with valvular surgery, cardiac surgery (eg, ventriculotomy, atriotomy) or valvular cardiac (surgical or percutaneous) procedure. - Unstable angina pectoris within the past 6 months. - Anticipated cardiac transplantation, cardiac surgery, or other major surgery within the next 12 months. - Significant pulmonary disease (eg, restrictive pulmonary disease, constrictive or chronic obstructive pulmonary disease) or any other disease or malfunction of the lungs or respiratory system that produces severe chronic symptoms. - Known significant PV anomaly that in the opinion of the investigator would preclude enrollment in this study. - Prior diagnosis of PV stenosis. - Pre-existing hemi diaphragmatic paralysis. - Acute illness, active systemic infection, or sepsis. - Presence of intracardiac thrombus, myxoma, tumour, interatrial baffle or patch or other abnormality that precludes catheter introduction or manipulation. - Severe mitral regurgitation. - Presence of implanted pacemaker or ICD or other implanted metal cardiac device that may interfere with the pulsed electric field energy. - Presence of a condition that precludes vascular access (such as IVC filter). - Significant congenital anomaly or a medical problem that in the opinion of the investigator would preclude enrollment in this study. - Categorised as vulnerable population and requires special treatment with respect to safeguards of well-being. - Current enrollment in an investigational study evaluating another device or drug. - Women who were pregnant (as evidenced by pregnancy test if premenopausal), lactating, or who were of child-bearing age and planned on becoming pregnant during the course of the clinical investigation. - Life expectancy <12 months. - Presenting contra-indications for the devices used in the study, as indicated in the respective instructions for use. |
| **Additional exclusion criteria for the subsets** | Additional exclusion criteria for neurological assessment patients:   - Contraindication for MRI such as use of contrast agents due to advanced renal disease, claustrophobia, etc (at principal investigator discretion). - Presence of iron-containing metal fragments in the body. - Unresolved pre-existing neurological deficit.   Additional exclusion criteria for esophageal endoscopy patients:   - Uncontrolled significant gastroesophageal reflux disease. |

AAD, antiarrhythmic drug; AF, atrial fibrillation; BMI, body mass index; ICD, implantable cardioverter defibrillator; IVC, inferior vena cava; LA, left atrial; LAD, left anterior descending; LVEF, left ventricular ejection fraction; MRI, magnetic resonance imaging; NYHA, New York Heart Association; PV, pulmonary vein; PVI, pulmonary vein isolation; TIA, transient ischaemic attack.

**Supplemental Table S3. Procedural information. Per protocol analysis set.**

| Procedural outcome | N = 136 |
| --- | --- |
| Sedation^a^ | 20 (14.7) |
| General anaesthesia | 116 (85.3) |
| Total procedure time, minutes | 108.0 (90.5, 126.0) |
| Catheter type used for LA map  Lasso  Pentaray  Octaray | 11 (8.1)  62 (45.6)  63 (46.3) |
| LA mapping time, minutes | 8.0 (7.0, 11.0) |
| Total fluoroscopy duration, minutes | 4.2 (2.2, 7.7) |
| Diagnostic fluoroscopy duration, minutes | 3.2 (1.7, 5.6) |
| Ablation fluoroscopy duration, minutes | 0.7 (0.2, 2.0) |
| DE STSF LA dwell time, minutes | 77.0 (64.0, 95.0) |
| Total PV ablation time, minutes | 54.0 (42.5, 66.0) |
| Total ablation duration, minutes^b^ | 57.9 (43.6, 75.0) |
| Total valid PF/RF application time, minutes^b,c^ | 12.8 (10.1, 18.7) |
| Number of valid PF/RF applications for PVI^c^ | 67.0 (58.0, 81.0) |
| RF; n=135 | 31.0 (25.0, 37.0) |
| PF; n=136 | 37.5 (28.0, 48.0) |
| Fluid delivered via the study catheter(s), mL; n=126^d^ | 400.0 (300.0, 500.0) |

Data are shown as n (%) or median (Q1, Q3).

CTI, cavotricuspid isthmus; DE STSF, THERMOCOOL SMARTTOUCH SF; LA, left atrial; PF, pulsed field; PFA, pulsed field ablation; PV, pulmonary vein; PVI, pulmonary vein isolation; Q, quartile; RF, radiofrequency.

^a^Sedation was performed with midazolam, fentanyl, and propofol, in accordance with the standard-of-care practice at 2 study sites.

^b^CTI ablation was included.
^c^PFA with all applications <100% status were excluded and considered as invalid; the time spent on energy delivery was counted as ablation time, excluding the intervals between each delivery.
^d^For 10 patients, fluid was delivered via the study catheter, but the amount was not registered by the site.

**Supplemental Table S4. Procedural parameters in patients with or without the VIZIGO sheath. Per protocol analysis set (n=136).**

|  | **Patients with VIZIGO** | **Patients without VIZIGO** | ***P* value** |
| --- | --- | --- | --- |
| Patient number | 52 (38.2) | 84 (61.8) |  |
| Total fluoroscopy duration (minutes) | 2.8 (1.7, 5.6) | 5.1 (3.3, 8.2) | <0.001 |
| Diagnostic fluoroscopy duration | 2.1 (1.3, 4.6) | 3.8 (2.3, 5.7) | 0.001 |
| Ablation fluoroscopy duration | 0.3 (0.1, 1.3) | 1.0 (0.3, 2.5) | 0.001 |
| Total procedure time (minutes) | 98.0 (75.0, 117.5) | 110.0 (94.5, 132.0) | 0.004 |

Data are shown as n (%) or median (Q1, Q3). *P* values were calculated by Wilcoxon rank-rum test.

**Supplemental Figure S1. Patient disposition.**

**
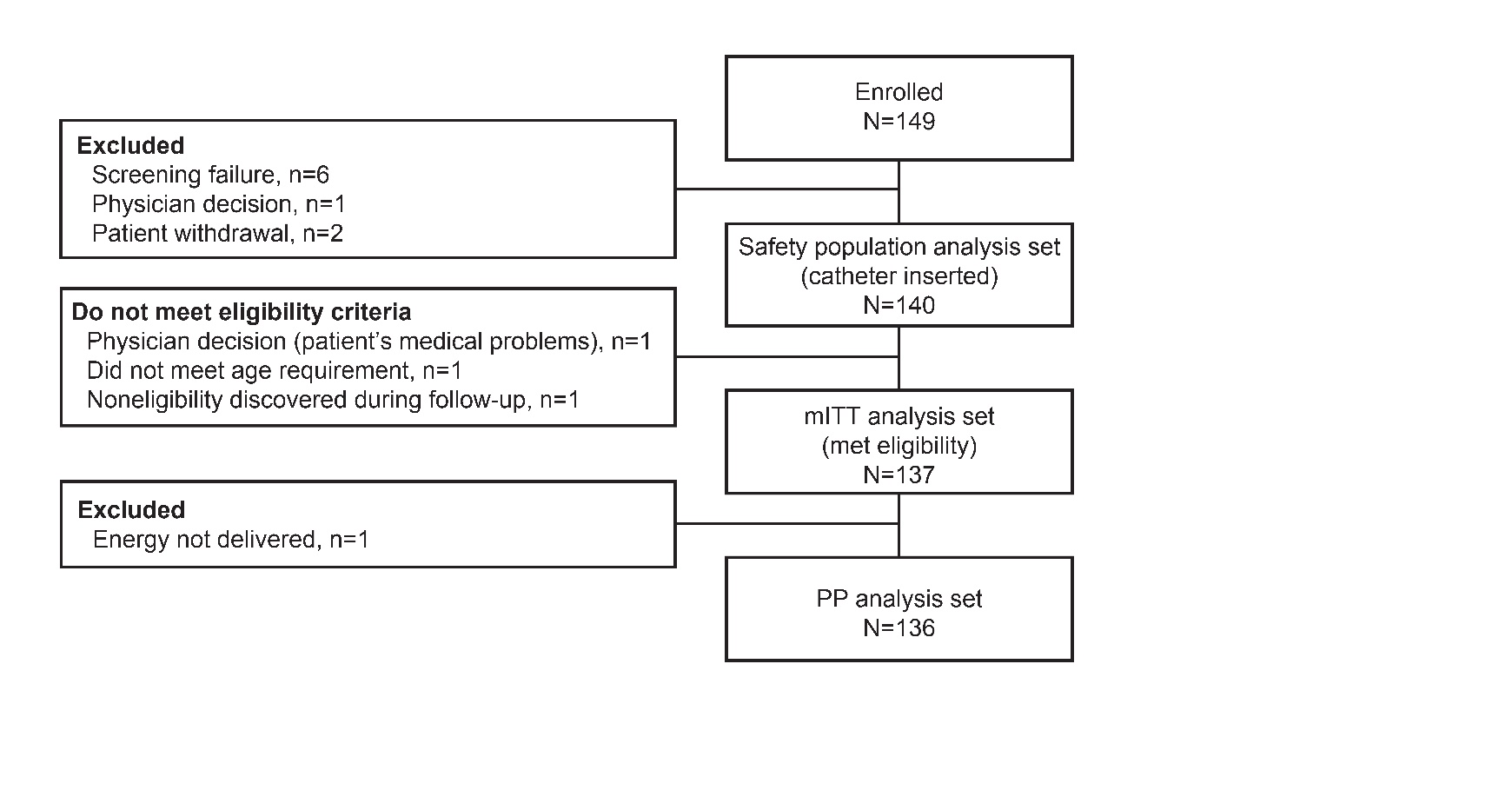
**

mITT, modified intent-to-treat; PP, per-protocol.

Images are courtesy of © Biosense Webster, Inc., part of Johnson & Johnson MedTech. All right reserved.

**Supplemental Figure S2. Index procedure of patients presenting with PV stenosis**


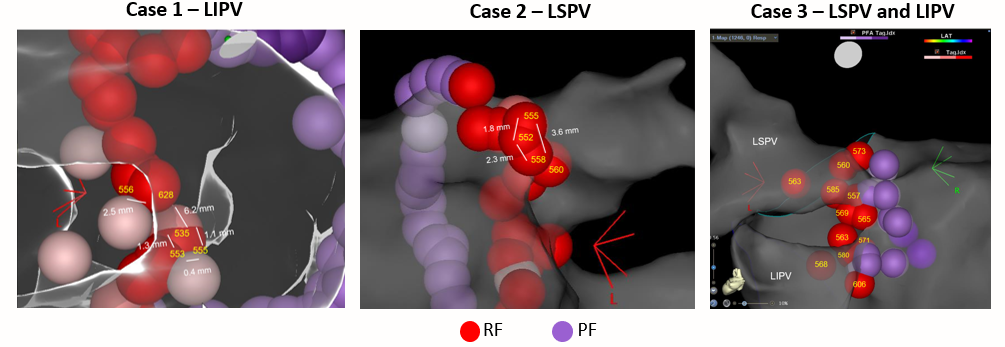


All PV stenoses occurred in the left PVs and were associated with ostial applications and/or excessive RF ablation (high RF index and overlapping lesions) during the index procedure.

LIPV, left inferior pulmonary vein; LSPV, left superior pulmonary vein; PF, pulsed field; PV, pulmonary vein; RF, radiofrequency.

Images are courtesy of © Biosense Webster, Inc., part of Johnson & Johnson MedTech. All right reserved.
